# Construction Notes

**Published:** 1894-07-07

**Authors:** 


### CONSTRUCTION NOTES.

### Foresters' Convalescent Home at Clent.

This is intended to accommodate thirty patients, and is in substitution of the home about half its size in the same neighbourhood. The new home has been built at a cost of £3,250, towards which donations have been received from 27,951 members. It should be stated that land has been acquired so that when the necessary support is forthcoming provision may be made in the home for sixty patients. The home is a plain Gothic building of red brick, relieved with stone dressings, and its cost has been about £2,000, while £300 has to be added for its equipment.

### Proposed Alterations at Huddersfield Infirmary.

A new wing is to be added to the above-mentioned Infirmary. This scheme will include the pulling down of the existing south wing, and the erection of a new three-tier wing in its place, to be connected with the main building with gangways only. This also will necessitate a change of locality for the laundry, which at present is situated in the south wing, immediately below the out-patient department. The addition will allow reorganisation of the whole out-patient and casualty department, will furnish a suite of operation rooms on the most modern principles on the third floor, together with wards for special and abdominal cases, two large wards, and increased sleeping accommodation for nurses and servants. Mr. B. Stocks, of Union Bank Yard, New Street, Huddersfield, is the architect.

